# Defining career success: A cross-sectional analysis of health information managers’ perceptions

**DOI:** 10.1177/18333583231184903

**Published:** 2023-07-25

**Authors:** Abbey Nexhip, Merilyn Riley, Kerin Robinson

**Affiliations:** 1 La Trobe University, Australia; 2 Goulburn Valley Health, Shepparton, Australia

**Keywords:** health information management, health information manager, health information management profession, health information management workforce, career success, objective career success, subjective career success

## Abstract

**Background:**

Career success can be defined as the accomplishment of desirable outcomes in an individual’s work experiences. It can be divided into objective and subjective career success. Objective success refers to tangible and measurable outcomes such as promotions and position titles. Subjective career success relates to an individual’s interpretations of their success or accomplishments. The career success of health information management professionals has not been explored in the literature.

**Aim:**

To determine the indicators of career success as reported by health information managers (HIMs) and identify whether there are any differences based on length of time in the profession.

**Methods:**

Using a cross-sectional study design, an online survey was administered to a sample of La Trobe University and Lincoln Institute of Health Sciences Medical Record Administration and Health Information Management graduates from 1985, 1995, 2005 and 2015, which included the following question: “How would you define success in your career?”

**Results:**

Almost 88% (*n* = 63) of overall participants in the study responded to this item. Subjective factors (*n* = 77) of career success, compared to objective factors (*n* = 22), were more common. The categories of recognition (feeling valued/appreciated), job satisfaction and feelings of accomplishment/sense of achievement were commonly reported.

**Discussion:**

Subjective factors of an individual’s career success were deemed to be more significant than objective factors among HIMs.

**Conclusion:**

Factors such as recognition and appreciation at work, job satisfaction, fostering high-quality work outputs and creating a sense of achievement should be the major foci for managers, organisations and individuals.

## Introduction

Career success has been broadly defined as the positive feelings that an individual attaches to their work-related outcomes, accomplishments and experiences (Converse et al., 2012; Ng et al., 2005). Two distinctive dimensions of career success have been identified: objective (or extrinsic) and subjective (or intrinsic) (Arthur et al., 2005; Ng et al., 2005; Spurk et al., 2018). Objective career success can be represented through calculable indicators of an individual’s career, such as income, promotions and attained organisational position (Arthur et al., 2005). Conversely, the subjective pillar is determined by an individual’s appraisal of their success within the context of their work experience(s), such as job satisfaction, work-life balance and a sense of accomplishment (Guan et al., 2019; Heslin, 2005; Stumpf and Tymon, 2012).

The careers landscape has changed significantly in the last 20 years to accommodate technological, societal, political, economic and organisational changes (Chudzikowski, 2012; Colakoglu, 2011). The cumulative adjustments have seen the subtle deconstruction of the traditional career, which was characterised by upward role attainment in a single organisation (Colakoglu, 2011). This employment pattern was considered a criterion for objective career success (Stumpf, 2014). In contrast, contemporary employees are more likely to pursue roles that they consider meaningful (Ng and Feldman, 2014) and are, therefore, less likely to anticipate a long-term career within a single organisation. This career concept has been described as the “protean career” (Converse et al., 2012: 149), where individuals are becoming more determined to shape their own careers and are open to flexible and dynamic movement between workplaces. This shift from a career conditional upon “linear” (Ng and Feldman, 2014: 169) mobility within one workplace, to a career path where transition may occur more frequently (Chudzikowski, 2012), has amplified the importance of subjective career success. This is consistent with the findings of several researchers who have argued that the subjective interpretation of one’s career is the primary indicator for career success, rather than objective measurements (Heslin, 2005; Shockley et al., 2016). Individuals are increasingly making career-related decisions based on subjective characteristics, rather than on factors such as salary or promotions. Contemporary career mobility, as described by Chudzikowski (2012), can be attributed to an increase in contractualised and project-based employment opportunities. All of these factors contribute to an increasingly complex working environment. This complexity can make it difficult for individuals to navigate their career paths and understand their feelings towards their work (Converse et al., 2012; Ng et al., 2005).

Achieving career success is valuable to both the individual and their organisation. It enhances an individual’s energy and dedication to their work and leads to continuous professional development, improved quality of work, increased staff retention rates and fewer errors (Dyess et al., 2015; Zhang et al., 2020). Ng et al. (2005) advised that understanding the determinants of career success can assist organisations in meeting their goals.

### Human capital theory

Several theories have served as a theoretical underpinning within the career success literature. In the current study, Becker’s (1993) human capital theory was used to underlie the concept of career success. Becker (1993) posited that every individual brings a set of different skills, education, experience and personal characteristics to their job. Shahibudin (2015) asserted that the amount of human capital a person utilises within their job (such as their effort and knowledge) is the main determining factor of career success. The current study aimed to highlight how health information managers (HIMs) define their career success which, in turn, may reflect their perceptions of their human capital.

### The link between motivation and career success

The career literature suggests that individuals who are self-determined in their work motivation will achieve high career success (Dahling and Lauricella, 2016; Schmitt et al., 2021). Schmitt et al. (2021) found that women in science, technology, engineering and mathematics who felt under-valued, and consequently demonstrated amotivation, perceived themselves as having lower levels of career success. Career success was described by Hupkens et al. (2021) as a dynamic concept that can be continuously (re)shaped and (re)assessed. For example, when the individual’s work-related goals are met, or no longer serve that individual, they will develop new career goals and internal motivators. Through this adaptive process, their perceptions of career success will continuously change to align with their goals (Hupkens et al., 2021). This was reinforced by McDonald and Hite (2008), for whom career success constitutes much more than verifiable measurements such as promotions and pay rises, but rather, is influenced by a range of factors. This links to the concept of motivation, which is continuously changing and based on internal and external influences (Dysvik and Kuvaas, 2013).

The way in which HIMs define success in their career is a topic that has not previously been reported in the literature.

## The current study

This research was part of a larger study that aimed to investigate the motivators of HIMs in the construction of their professional identity and associated relationships to job satisfaction and engagement with their profession. The objectives of this component of the research were to

(1) Determine the subjective and objective factors of career success as reported by HIMs; and,
(2) Identify whether there are any differences in factors that impact on career success, based on length of time in the profession.

## Method

### Study design and sample

A cross-sectional study design using a convergent mixed-methods approach (Creswell and Creswell, 2017) was utilised. Data were collected from the 1985, 1995, 2005 and 2015 Medical Record Administration and Health Information Management graduate cohorts from La Trobe University (LTU) and Lincoln Institute of Health Sciences, in the state of Victoria. The method has been reported previously (Nexhip et al., 2022).

Graduates from the requisite cohorts were approached using three recruitment methods, via information contained in an LTU Discipline of Health Information Management database; LinkedIn; or the researchers’ professional networks. Graduates who had retired or changed careers from health information management to another profession were included. Exclusions included graduates whose contact details could not be obtained, who had died or who were a member of the research team. Of the 136 eligible graduates, a sample of 99 was reached following application of the exclusion criteria.

### Data collection

An online Research Electronic Data Capture (REDCap) survey was developed to collect HIMs’ responses regarding motivation; engagement with their profession; job satisfaction; professional identity and reflection on professional careers. In the open-ended items eliciting qualitative data about motivation, the following was asked: “How would you define success in your career?” The responses for this component of the survey are reported herein.

### Data analysis

The data were analysed using qualitative content analysis. The free-text responses on how HIMs defined success in their career were systematically grouped into specific content categories (Hesse-Biber and Leavy, 2004). This was performed by a single researcher (AN), with verification completed by the remainder of the research team. The categories of career success indicators identified by Hupkens et al. (2021) were used (in a customised format) to categorise the qualitative data (Box 1).

**Box 1.** Overview of career success aspects and their explanation.

Career success aspect	Explanation
Subjective (intrinsic) factors	
Autonomy	This is characterised by an individual’s feeling of personal ownership towards their career, such as being responsible for their own projects (Hupkens et al., 2021).
Personal development	The attainment of knowledge and skills in order to maintain professional competence and improve work performance (Shockley et al., 2016).
Influence	Having an impact on an organisation through seeing the outcomes of personal influence (i.e. decision making and actions) (Hupkens et al., 2021).
Service to others	The feeling of contributing to something greater than the individual; engaging in meaningful work (Hupkens et al., 2021).
Work–life balance	The balance between work and non-work responsibilities. A career where an individual feels capable of successfully maintaining energy and time for both work, and life outside of work (Hupkens et al., 2021).
Quality work	High-quality work outputs and performance; feeling fulfilled by performing well (Hupkens et al., 2021).
Recognition	The feeling of being valued, appreciated and recognised within a professional role (Hupkens et al., 2021).
Career satisfaction	An overall subjective evaluation of a person’s satisfaction with their career. When an individual is generally satisfied with their work (Hupkens et al., 2021).
Positive relationships	The quality of the relationships with colleagues and managers in the social context of an individual’s career (Hupkens et al., 2021).
Objective (extrinsic) factors	
Financial security	Being able to provide basic living needs (Hupkens et al., 2021).
Financial success	Receiving a higher salary throughout the course of one’s career (Hupkens et al., 2021).

### Ethics approval

The study was approved by the LTU Human Research Ethics Committee.

## Results

### Response rate

As reported previously by Nexhip et al. (2022), 72 responses were received from 99 (72.7%) invitations to complete the survey. Sixty-three (87.5%) respondents answered the item addressing career success. Table 1 highlights the response rate, by graduate cohort.

**Table 1.** Response rate for the question: “How would you define success in your career?”

Cohort	Number of responses to question	Number of responses to survey	Response rate (%)
1985	10	10	100
1995	11	12	91.7
2005	11	15	73.3
2015	31	35	88.6
Total	63	72	87.5

### Indicators of career success

**Figure 1.**
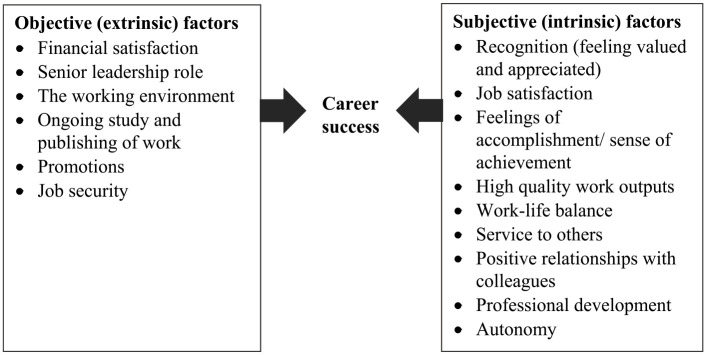
Factors in objective and subjective career success of health information managers (HIMs).

Figure 1 summarises the key indicators of career success, grouped into the broader categories of objective and subjective factors, using a modified version of those identified by Hupkens et al. (2021) (Table 1).

### Objective (extrinsic) career success

Table 2 highlights the categories, frequency and participants’ quotations relating to the objective factors of career success. Financial satisfaction (*n* = 5) and attaining a position in a senior leadership role (*n* = 5) were the most common objective factors indicative of a successful career.

**Table 2.** Quotations relating to HIMs’ objective career success.

Factor	Frequency	Quotation(s)
Financial satisfaction	5	• “. . . high salary” [17, 2015]. • “. . . a good income” [67, 1985]. • “. . . and renumeration” [15, 2015]. • “. . .I have hours and pay that suit me. . .” [4, 2015]. • “high salary. . .” [19, 2015].
Senior leadership role	5	• “New grad[uate] to department head in 7 years” [8, 1985]. • “A senior leadership role, which I am in now. . .” [14, 1985]. • “Being in a management role that is responsible for staff” [62, 2015]. • “. . .achiev[ing] higher level positions” [63, 2005]. • “Leading a team, leveraging their skillsets to ensure the centrality of HIMs to the future of healthcare funding” [69, 2015].
The working environment	4	• “. . . and working in a friendly environment” [19, 2015]. • “. . . and also employed in a well-supported health organisation” [13, 2015]. • “Having a happy team and pleasant work environment” [32, 1995]. • “A positive workplace culture. . .” [56, 2015].
Promotions	3	• “Reaching a grade 3 position” [51, 2015]. • “Being considered for promotions” [32, 1995]. • “Promotions. . .” [33, 2015].
Job security	3	• “Keeping a steady permanent role in a supportive organisation” [37, 2015]. • “Reliable, permanent employment” [35, 2015]. • “The opportunity to work continuously throughout 20+ years in a variety of HIM related roles” [22, 1995].
Ongoing study and publishing of work	2	• “High reputation (number of article[s], hold[ing] a high degree such as PhD)” [19, 2015]. • “I have continued to undertake post-grad[uate] study throughout my career and feel very satisfied when I am studying” [20, 1985].
Total	22	

HIM: health information manager.

*Note*: [xx, yyyy] indicates respondent ID and year of graduation, respectively.

### Subjective (intrinsic) career success

Table 3 highlights the factors of subjective career success and their frequency, with related quotations from participant HIMs.

**Table 3.** Quotations relating to HIMs’ subjective career success.

Factor	Frequency	Quotation(s)
Recognition (feeling valued and appreciated)	15	*Recognised and respected by co-workers* • “. . .recognised by managers and leadership level” [63, 2005]. • “. . .from my managers as being an expert in my field” [46, 2015]. • “Engagement and recognition from other staff within organisation that you work” [41, 1995]. • “. . .source of information for younger or newer employees. . .” [8, 1985]. • “Becoming a go-to for information, trusted source” [26, 2015]. • “Respect from co-workers, being the ‘go to’ subject matter expert” [42, 1985]. • “I feel valued and challenged. People consult me about issues involving systems, processes, new tools, new ideas” [20, 1985]. • “Success in my career would be defined as [being] respected by my peers, and known for being an approachable, engaging and supportive manager” [70, 1995]. *Feeling valued and appreciated by the organisation* • “. . .being valued by the organisation you work for” [2, 1985]. • “Acknowledgement for my role/projects” [71, 2015]. • “Being known as a source of reliable ‘help’ and a ‘go-to’ person within my organization. Respected” [16, 1995]. • “Knowing that my work and I am appreciated” [57, 2005]. • “A career where I am appreciated. . .” [5, 2015]. *Acknowledgement among the health information management profession* • “Professional recognition and contribution to the professional community” [10, 2015]. • “Professional admiration. It is a small community in the HIM world. You don’t want a bad reputation or to be known as a weak coder” [23, 1995].
Job satisfaction	14	• “A career where I am . . . feeling satisfied” [5, 2015]. • “Job satisfaction and happiness” [6, 2015]. • “Feeling fulfilled and satisfied with the tasks in my role while being challenged to grow and develop” [34, 2015]. • “Being happy in my role, enjoying the work that I do. . .” [39, 2015]. • “I would define success in my career being happy and enthusiastic when I do my work. I can be tired but I never felt that I don’t want to do this work. . .” [13, 2015]. • “Holistic and equal satisfaction across the facets of the work itself. . .” [15, 2015]. • “Be[ing] able to work in an area that I enjoy” [36, 2005]. • “. . . and overall satisfaction” [56, 2015]. • “Being [in] a position that I enjoy and love” [65, 2005]. • “. . .job satisfaction. . .” [67, 1985]. • “Working in an area of health information that is of interest to me because that is why I studied HIM” [1, 2015]. • “Successfully supporting my family with a career that has a level of challenge and satisfaction” [53, 1995]. • “Clinical coding suits me better than any other job I’ve ever worked in . . . I am interested in my work” [4, 2015]. • “Job satisfaction” [38, 2015].
Feelings of accomplishment/sense of achievement	13	• “Achieving my goals and feeling that I am making a difference” [7, 1985]. • “I believe in looking back to identify achievements” [50, 2005]. • “I define success as a combination of personal and professional developments in short-term goals (mini projects, process developments) and long-term goals (organisational strategies and goals)” [52, 2015]. • “Doing what I need to do with the best of my abilities” [11, 2015]. • “Success in my career would be defined as achieving goals I have set for myself. These goals evolve over time, but are short term and long-term goals” [70, 1995]. • “daily achievement of job well done” [55, 1985]. • “feelings of accomplishment” [3, 1985]. • “To be able to look back and see how far I have come in my management career” [43, 2015]. • “. . .and I feel that as I gain more experience I am improving/becoming a better coder. This is all success to me” [4, 2015]. • “Being resilient as being a HIM you have many different skillsets which provide many opportunities and challenges” [31, 2015]. • “Keeping a . . . role . . . that provides growth and challenges me” [37, 2015]. • “. . .feeling challenged/inspired to improve not only myself but others around me” [39, 2015]. • “Good feedback from management and colleagues” [35, 2015].
High-quality work outputs	11	• “No DRG/no coding change. Maybe become a member of VICC” [60, 2015]. • “Good audit results and improvement in other coders” [72, 2005]. • “Getting excellent results in the quality and quantity of coded data” [47, 2005]. • “Making information accessible as soon as its needed. Delivering projects on budget and on time within specifications that users want, need and expect” [21, 1995]. • “Always making sure deadlines are met” [44, 2015]. • “I feel that you can be successful in a role through the delivery of outcomes. . .” [45, 1995]. • “Meeting government deadlines and guidelines” [27, 1995]. • “Good outcomes” [2, 1985]. • “Achieving mandated work requirements. . .” [18, 2015]. • “. . .backed by evidence of managing high performing teams” [66, 2005]. • “Being able to engage with executive teams in a meaningful way that allows HIMS and Coders to participate in EMR and other technological developments in healthcare data collection and access” [69, 2015].
Work-life balance	6	• “Finally, I’d say that success is working in a role that has a manageable workload and doesn’t negatively affect my mental health” [1, 2015]. • “A career where I am . . . able to maintain a work/life balance” [5, 2015]. • “. . .where you can reach the balance of income, lifestyle and work is enjoyable and that it doesn’t feel like hard work” [45, 1995]. • “. . .managing children/family with work part time” [8, 1985]. • “I have work/life balance as I work part-time and flex my hours to meet workplace needs as they ebb and flow. This suits me and my employer” [20, 1985]. • “balanced home and work life. . .” [67, 1985].
Service to others	6	• “Making a difference and helping my organisation achieve their goals” [12, 2015]. • “Ability to be a team player and to mix well in order to provide a quality service” [29, 1995]. • “I endeavour to implement change where necessary to facilitate best practice across our hospital’s Health Information and Coding Services” [70, 1995]. • “Feeling as though my work has value and makes a difference in the lives of the patients we treat” [30, 2005]. • “. . . delivering successful IT projects to private and public healthcare organisations which enable and improve better health information exchange which in turn hopefully improves clinical outcomes for patients – is a good definition to me of success in my career. Doing my part to enable better healthcare” [9, 1985]. • “Demonstrating the role of HIMs as a crucial element of leadership within services to support both safe patient care and an effective transition to a digital environment” [25, 2015].
Positive relationships with colleagues	4	• “. . . the people you work with” [15, 2015]. • “Clinical coding suits me better than any other job I’ve ever worked in, I work with people I like and respect. . .” [4, 2015]. • “. . .and building working relationships with other HIMs” [18, 2015]. • “Driven, team player” [54, 2005].
Professional development	4	• “Being in a role that allows me to utilise my health information management background and continuously build upon my skills” [1, 2015]. • “Constant learning and professional development” [10, 2015]. • “Further development (never stop learning)” [32, 1995]. • “Attending personal development opportunities . . . while expanding out into special interest areas such as staff training” [18, 2015].
Autonomy	4	• “Clinical coding suits me better than any other job I’ve ever worked in . . . I am able to work independently but have the support of the team. . .” [4, 2015]. • “I do not want to manage a large team and I enjoy the autonomy and challenges that my work brings, with the ability to prioritise my own workload” [20, 1985]. • “. . . autonomy [and] opportunities for extra projects” [33, 2015]. • “A positive workplace . . . with flexibility [and] opportunities for variety of tasks/roles” [56, 2015].
Total	77*	

HIM: health information manager.

*Note*: [xx, yyyy] indicates respondent ID and year of graduation, respectively.

* The total number of responses is greater than the number of participants as some participant responses contained two or more components that were categorised into different factors.

Participants more commonly reported on subjective factors (*n* *=* 77), compared to objective factors (*n* = 22). The most common intrinsic responses from participants, when defining their career success, were embedded within the subjective categories of recognition (feeling valued and appreciated) (*n* = 15), job satisfaction (*n* = 14) and feelings of accomplishment/sense of achievement (*n* = 13).

Respondents acknowledged the receipt of recognition in three ways: feeling valued and appreciated by the organisation; being recognised and respected by co-workers and being acknowledged within the health information management profession. Respondents commented on the attainment of being a “subject matter expert” [42] and a “go-to for information” [26] within their workplace as an indicator of career success. Job satisfaction, as a driver of career success, was reported by approximately 22% (*n* = 14) of participants. Respondents commented on “enjoying the work that [I] do” [39] and “being happy and enthusiastic . . . [at] work” [13] as definitions of work-related career success. The third most common factor of career success was related to intrinsic motivation and achievement of work-related goals. Respondents measured their career success according to “feelings of accomplishment” [3] and “feeling challenged/inspired to improve. . .” [39].

## Discussion

This study has provided significant insight into the perspectives on career success of cross-generational cohorts of health information management graduates. The results support Heslin’s (2005) and Shockley et al.’s (2016) notion, that subjective factors of career success are deemed to be more meaningful than objective factors. The study has identified that the major factors of career success relate to the respondent HIMs’ appreciation by managers and colleagues, job satisfaction, intrinsic feelings of accomplishment and production of high-quality work that is specific to their role.

### Subjective factors of HIMs’ career success

The results demonstrate that the feeling of being valued and appreciated at work was the primary subjective factor in how HIMs in this study defined success in their career. The concept of appreciation at work relates to the acknowledgement of an individual’s values, achievements and personal characteristics in a positive manner (Semmer et al., 2016). Appreciation at work has also been addressed by Van Quaquebeke et al. (2009) within the context of appraisal and recognition respect; that is, the respect employees receive for their work, from supervisors and colleagues, as well as the respect they receive as a person. Receiving respect in the workplace enhances an individual’s self-esteem and can lead to higher perceptions of career success and satisfaction (Van Quaquebeke et al., 2009). Takeuchi et al.’s (2008) study of physiotherapists found that recognition from their patients and colleagues for their efforts was a marker for career success, and although HIMs are not directly involved in patient interactions, our findings highlighted the intrinsic value of recognition from colleagues in a health context. Reinforcing this, approximately 24% (*n* = 15) of the responses to this question in the current study identified the feeling of being valued and appreciated at work as an important element of success in the HIMs’ roles. This was reported by members of all cohorts, suggesting that the importance of being recognised for the work that HIMs do is significant across their early-, mid- and late-career stages.

The general satisfaction that an individual has within their job is another important factor of career success. The feeling of “being happy in [my] role” [39] was reported in 22% (*n* = 14) of the responses. The nexus between job satisfaction and career success was examined by Tremblay et al. (2014), who found that individuals experiencing job success feel a greater sense of satisfaction with their roles and have a greater appreciation of their employing organisation. In their study of professionals working within the technology sector in Canada, Dyke and Duxbury (2010) found that enjoyment of work and a personal sense of accomplishment were the two most important factors of career success. The latter was also identified as an important factor of career success in the current study, with approximately 21% (*n* = 13) of the responses reporting “achieving goals” [70] or other feelings of personal accomplishment as definitions of HIMs’ work-related success. This is consistent with findings from other components of this study: in the context of McClelland’s (1985) achievement needs theory, HIMs are inherently motivated by the desire to fulfill and accomplish set goals (Nexhip et al., 2022).

### Objective factors of HIMs’ career success

Financial satisfaction and attained organisational position emerged from the objective factors (related to the organisation or external to the individual) as the most common examples of career success. While not as common as subjective factors (*n* = 77), objective factors of career success were reported 22 times by participants in the current study. This is consistent with the findings of Abele et al.’s (2010) study of university-qualified professionals from a range of disciplines, specifically that their perceptions of career success and satisfaction were largely based on factors external to money and position.

### Practical implications

From a practical perspective, the results of this study underscore the importance of subjective factors in shaping an individual’s perceptions of their career success, with a particular focus on appreciation and recognition at work. They suggest implications for individuals and organisations. The greater acknowledgement of subjective factors indicates that individuals are increasingly occupying roles that offer compatibility with their personal lives and values (Heslin, 2005; Shockley et al., 2016). This has led researchers to suggest that organisations and managers need to understand that salaries and promotions are not as important to an individual’s work-related success, in contrast to their internal feelings of being acknowledged, respected, satisfied and motivated to perform well in their work. This further supports the idea that individuals, when crafting their own careers, and organisations, when strategising employee engagement and retention, should pay more attention to the subjective meanings of career success.

### Limitations

Measurement of career success was not the primary focus of the main study; therefore, this concept was not captured using quantifiable measurement tools. Responses to the open-ended item were informative in eliciting information on how the HIMs defined success in their careers; however, the integration of a quantifiable career success measurement tool would enrich the findings in this area. Additionally, the relative numbers in each graduate cohort suggest a higher representation of early-career respondents in the study. The sample of Victorian graduates is a limitation, and further studies on a wider cohort of Australian HIMs would enhance generalisability of results and allow for cross-jurisdictional analyses. Nexhip et al. (2022) have previously reported the study limitations more extensively.

## Conclusion

The “work world” continues to change and adapt in response to technological, societal and economic shifts. Consequently, individuals have an increased responsibility to self-manage their careers, which can make it difficult for them to orientate their work-related preferences and pathways. The findings of the current study highlighted the subjective and objective dimensions of how the HIM respondents defined success in their careers. The results suggested that meeting employees’ aspirations for success may be best achieved through subjective factors such as feelings of accomplishment, career satisfaction, high-quality work outcomes, work-life balance, and the achievement of work-related goals, as opposed to objective factors such as salary and promotions. No trends could be identified in factors that were more prominent, based on length of time in the profession, implying that subjective elements of HIMs’ career success were commonplace regardless of the individual HIM’s position in early-, mid- or late-career stages. The findings have led the researchers to recommend that HIMs with staff management portfolios should nourish subjective career success factors in their management approaches. In an environment where individuals are increasingly required to take ownership of their career development, health information management professionals should be proactive and consider which factors are the most important in their work-related success. Understanding this can lead to higher levels of productivity, better quality outputs and increased employee engagement: benefits for both individuals and employing organisations.
